# Soluble CTLA-4 attenuates T cell activation and modulates anti-tumor immunity

**DOI:** 10.1016/j.ymthe.2023.11.028

**Published:** 2023-12-05

**Authors:** Paul T. Kennedy, Emma L. Saulters, Andrew D. Duckworth, Yeong Jer Lim, John F. Woolley, Joseph R. Slupsky, Mark S. Cragg, Frank J. Ward, Lekh N. Dahal

**Affiliations:** 1 Department of Pharmacology and Therapeutics, University of Liverpool, L69 3GE Liverpool, UK; 2 Department of Molecular and Clinical Cancer Medicine, University of Liverpool, L69 3GE Liverpool, UK; 3 Centre for Cancer Immunology, University of Southampton, SO16 6YD Southampton, UK; 4 Department of Immunology, University of Aberdeen, AB25 2ZD Aberdeen, UK

**Keywords:** soluble CTLA-4, cancer, immunomodulation, immune checkpoint, immune regulation, immunotherapy

## Abstract

CTLA-4 is a crucial immune checkpoint receptor involved in the maintenance of immune homeostasis, tolerance, and tumor control. Antibodies targeting CTLA-4 have been promising treatments for numerous cancers, but the mechanistic basis of their anti-tumoral immune-boosting effects is poorly understood. Although the *ctla4* gene also encodes an alternatively spliced soluble variant (sCTLA-4), preclinical/clinical evaluation of anti-CTLA-4-based immunotherapies have not considered the contribution of this isoform. Here, we explore the functional properties of sCTLA-4 and evaluate the efficacy of isoform-specific anti-sCTLA-4 antibody targeting in a murine cancer model. We show that expression of sCTLA-4 by tumor cells suppresses CD8^+^ T cells *in vitro* and accelerates growth and experimental metastasis of murine tumors *in vivo*. These effects were accompanied by modification of the immune infiltrate, notably restraining CD8^+^ T cells in a non-cytotoxic state. sCTLA-4 blockade with isoform-specific antibody reversed this restraint, enhancing intratumoral CD8^+^ T cell activation and cytolytic potential, correlating with therapeutic efficacy and tumor control. This previously unappreciated role of sCTLA-4 suggests that the biology and function of multi-gene products of immune checkpoint receptors need to be fully elucidated for improved mechanistic understanding of cancer immunotherapies.

## Graphical abstract

**Figure:**
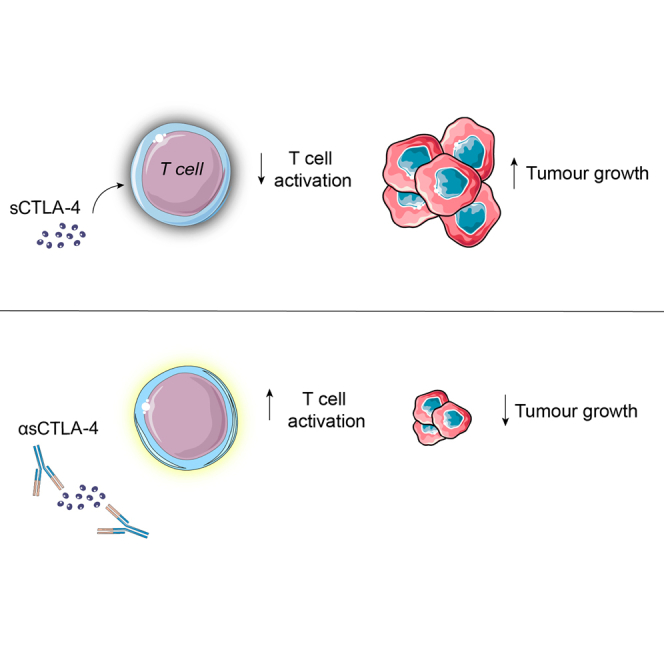

## Teaser

The role of soluble CTLA-4 in the immune regulation of cancer is poorly understood. In this study, Kennedy et al. identify a novel checkpoint function of this isoform, revealing it to suppress anti-tumor immunity and leverage therapy through its blockade.

## Introduction

Cytotoxic T lymphocyte-associated antigen 4 (CTLA-4) is a regulator of T cell activation and was the first molecule successfully targeted for immune checkpoint therapy.^1^ However, the mechanisms by which it suppresses immune responses *in vivo* remain ill defined.^2^^,^^3^ Constitutively expressed as a membrane protein on the surface of regulatory T (T_reg_) cells and activated effector T cells, CTLA-4 competes with CD28 for engagement of the B7 ligands CD80/CD86 on antigen-presenting cells (APCs). Such interaction reverses CD28-mediated co-stimulation of T cells via a mechanism involving induction of negative signal transduction^4^ and is responsible for controlling autonomous activation of T cells^5^^,^^6^ as well as cell-extrinsic regulation of distal T cell populations.^7^^,^^8^ Thus, our current understanding of how CTLA-4 functions centers around its membrane-bound isoform structure.^9^ Here it is important to note that *CTLA4* transcripts in humans and mice can be alternatively spliced to yield membrane-bound and secreted variants, the latter resulting from deletion of the exon encoding the transmembrane domain and a frameshift giving rise to a unique C-terminal sequence.^10^^,^^11^ This is important because currently available anti-CTLA-4 antibodies, including those used clinically, do not distinguish between the membrane-bound and soluble isoforms of CTLA-4 (sCTLA-4), limiting the scope for unraveling the contribution of sCTLA-4 in functional and therapeutic studies.

Several hypotheses have been proposed to explain the mechanisms of anti-CTLA-4 immunotherapy in cancer. These include deletion of intratumoral T_reg_ cells,^12^ modulation of T cell receptor (TCR)-CD28 interaction,^13^ and regulating naive T cell activation and differentiation.^14^ However, variation in clinical response is observed, and some studies have suggested that sCTLA-4 secreted by tumor cells may be responsible. For example, elevated levels of sCTLA-4 are observed in serum from patients with malignant melanoma,^15^^,^^16^ mesothelioma,^17^ and acute B cell lymphoblastic leukemia.^18^ A retrospective analysis of a small cohort of metastatic melanoma patients demonstrated that patients with higher levels of sCTLA-4 levels were more likely to respond to the anti-CTLA-4 antibody ipilimumab than those with lower levels.^19^ Although such correlative studies provide support for the clinical relevance of sCTLA-4 in cancer, the functional properties and feasibility of isoform-specific antibody targeting of this molecule for cancer immunotherapy remain to be fully explored.

Following the generation and characterization of selective anti-sCTLA-4 monoclonal antibodies raised against the unique C-terminal epitope of sCTLA-4, we have shown that it regulates certain cell-extrinsic aspects of CTLA-4 function associated with distal control of T cell effector responses in both health and disease.^20^^,^^21^^,^^22^^,^^23^ Contrary to previous assumptions, sCTLA-4 is produced as part of the natural immune response and should therefore be considered an important candidate regulatory mediator.^15^^,^^24^ Here, for the first time, we provide functional evidence of the strong immunosuppressive activity of sCTLA-4 *in vitro* and *in vivo* and demonstrate that it can be effectively targeted by an isoform-specific antibody to elicit anti-tumor activity.

## Results

### sCTLA-4 constrains T cell activation *in vitro*

**Figure 1:**
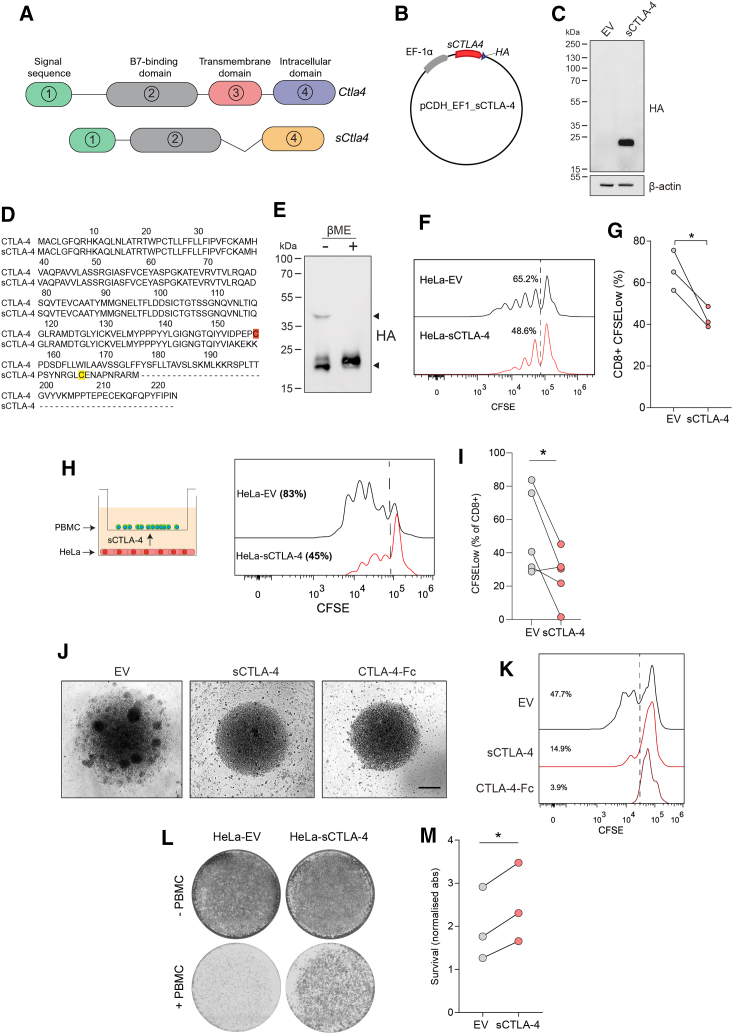
sCTLA-4 suppresses T cell activation and tumor cell killing *in vitro* (A) Alternative splicing of CTLA-4 gives rise to a soluble form of CTLA-4. Soluble CTLA-4 (sCTLA-4) retains the B7 binding domain encoded by exon 2 of membrane CTLA-4 but is missing the transmembrane domain encoded by exon 3. A reading frameshift during splicing gives rise to an alternative amino acid sequence encoded by exon 4. Both isoforms contain exon 1, which encodes a leader peptide. (B) Vector design for stable overexpression of recombinant sCTLA-4 in HeLa cervical adenocarcinoma cells. (C) Immunoblot showing transfected HeLa cells (HeLa-sCTLA-4 versus empty vector [EV]) under reducing conditions. (D) Alignment of human CTLA-4 and sCTLA-4 coding sequences. The membrane-proximal cysteine residue present in the transmembrane domain of membrane-bound CTLA-4 is highlighted in red. Although the transmembrane domain is absent in sCTLA-4, the C terminus in sCTLA-4 encodes another cysteine residue, highlighted in yellow. (E) Supernatant from HeLa-sCTLA-4 cells was immunoblotted under non-reducing and reducing conditions (−/+ βME). (F and G) Flow-cytometric analysis of CFSE-stained PBMCs stimulated with anti-CD3 following co-culture with HeLa-sCTLA-4 or EV HeLa cells (1:10 Hela-PBMC ratio). Histograms indicate CD8^+^ T cell proliferation after 4 days of stimulation. Data represent three independent PBMC donors (∗p < 0.05 Student’s t test). (H and I) Flow-cytometric analysis of CFSE-stained stimulated PBMCs in transwell co-cultures with HeLa cells. Histograms indicate CD8^+^ T cell proliferation after 4 days. Data represent five independent PBMC donors (∗p < 0.05, Student’s t test). (J) Splenocyte-BMDM co-cultures following stimulation with PMA/ionomycin and treatment with recombinant CTLA-4-Fc or sCTLA-4-conditioned medium (scale bar, 50 μm), representative of n = 3. (K) Flow-cytometric analysis of CD8^+^ T cells from (H); histograms indicate CD8^+^ T cell proliferation after 4 days. (L and M) T cell-mediated tumor cell-killing assay of HeLa-sCTLA-4 cells. Images show crystal violet-stained viable HeLa cells following co-culture with anti-CD3 activated PBMCs. Data represent three independent PBMC donors (∗p < 0.05, Student’s t test).

Gene variants (Figure 1A) of CTLA-4 have been reported to have utility as predictive biomarkers for anti-CTLA-4 therapy, improved long-term survival, and prediction of immune-related adverse events.^15^^,^^19^ These correlative studies were largely based on circulating serum levels of sCTLA-4, but the relevance and potential impact of sCTLA-4 mRNA expression in tumor tissue has not been investigated. Interrogation of publicly available bulk RNA sequencing (RNA-seq) datasets from The Cancer Genome Atlas^25^ revealed relative expression of membrane-bound versus sCTLA-4 within lung adenocarcinoma (LUAD) and skin cutaneous melanoma (SKCM) (Figure S1). In this analysis we found that the membrane-bound isoform was the more abundant RNA species in both tumor types, while there was a positive correlation in the expression level of each isoform that was greater in SKCM than in LUAD. Thus, tumor cells from patients express sCTLA-4 mRNA where the produced protein may modulate anti-tumor immune responses. To understand the functional capacity of sCTLA-4 to modulate anti-tumor T cell responses, we constructed expression vectors to generate tumor cells that constitutively secrete recombinant sCTLA-4 and created stable cell lines (Figures 1B and 1C) but which, importantly, do not express surface CTLA-4 (Figure S2). sCTLA-4 has been predominantly reported as a monomeric entity due to the splicing of exons 2 and 4 and the subsequent loss of a membrane-proximal cysteine residue at position 157.^11^^,^^26^ This cysteine residue, present in the membrane-bound CTLA-4, is presumed crucial for homodimerization, stable interaction with B7 ligands, and, thus, potent immunosuppressive properties.^27^^,^^28^ However, the skipping of exon 3 in sCTLA-4 results in a reading frameshift encoding an alternative cysteine residue which may also permit sCTLA-4 dimerization (Figure 1D).^11^ Indeed, we found a higher molecular weight component, consistent with the formation of a disulfide bridge between the *de novo* encoded cysteine residues resulting from the alternative splicing that generates sCTLA-4, indicative of dimeric sCTLA-4, in cell-culture supernatants from HeLa-sCTLA-4 cells, which under reducing conditions disassociated to form a monomer with the expected mass of ∼20 kDa (Figure 1E). The presence of a slightly higher band in the supernatant compared to lysate (Figure S3A) was shown to be the result of glycosylation differences, normalized by PNGase F treatment (Figure S3B), indicating that sCTLA-4 is possibly secreted as a glycosylated protein.

We next tested the immunosuppressive potential of sCTLA-4 on T cell activation. HeLa-EV (empty vector control) or HeLa-sCTLA-4 cells were co-cultured directly (Figures 1F and 1G) or in transwell setting (Figures 1H and 1I) with healthy donor human peripheral blood mononuclear cells (PBMCs) stimulated with anti-CD3 antibody. In both systems, CD8^+^ T cells exhibited reduced proliferation in the presence of sCTLA-4-secreting HeLa cells compared to those cultured with HeLa-EV control cells. A similar assay was performed using murine splenocytes treated with murine sCTLA-4-enriched supernatant. As a positive control within this assay we included a murine equivalent of Abatacept, which is a soluble recombinant CTLA-4-Fc fusion protein. Splenocytes stimulated with phorbol 12-myristate 13-acetate (PMA)/ionomycin displayed clear clusters of proliferating cells that were suppressed when they were co-cultured either with murine sCTLA-4 or with CTLA-4-Fc (Figure 1J). Furthermore, in keeping with our observations of human T cell responses, specific examination of murine splenic CD8^+^ T cells showed that the presence of murine sCTLA-4 or CTLA4-Fc reduced their proliferation compared to control cells (Figure 1K). Taken together, these results show that sCTLA-4 is functionally similar to the artificially engineered CTLA4-Fc and suppresses T cell proliferation and clustering.

Another aspect we investigated is whether sCTLA-4-induced constraints on T cell activation translated into a survival advantage for tumor cells *in vitro*. Accordingly, HeLa cells were stained and visualized post co-culture with activated PBMCs, revealing a markedly higher density of HeLa-sCTLA-4 cells than HeLa-EV cells, thereby indicating an enhanced resistance of target tumor cells to T cell killing in the presence of sCTLA-4 (Figures 1L and 1M). Indeed, this effect was preserved in co-cultures where natural killer (NK) cells were depleted from PBMCs, indicating that cytotoxicity against tumor cells in this co-culture system is primarily driven by T cells (Figure S4). These data, taken together with our determined functional role of sCTLA-4, suggest that tumor release of sCTLA-4 gives malignant cells a survival advantage by suppressing immune cell responses. Moreover, since sCTLA-4 is evolutionarily conserved in mammals,^11^ the results from this section further suggest that human response to sCTLA-4 can be accurately modeled in mice.

### sCTLA-4 promotes syngeneic tumor growth *in vivo*

**Figure 2:**
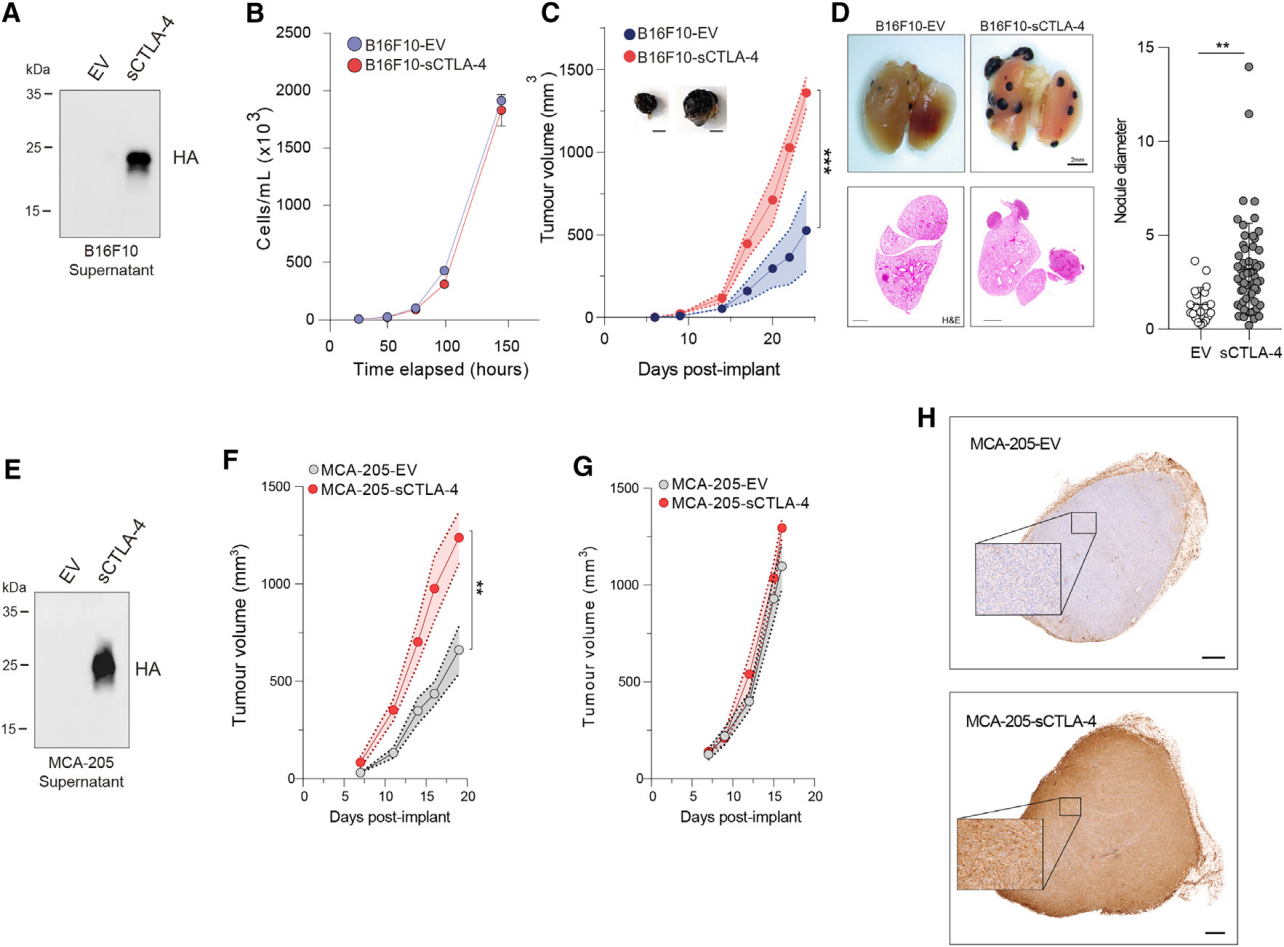
sCTLA-4 promotes syngeneic tumor growth *in vivo* (A) Immunoblot showing stable sCTLA-4 overexpression in B16F10 melanoma cells. *In vitro* (B) and *in vivo* (C) growth curves of B16-EV versus B16-sCTLA-4 cells. *In vivo* tumor growth data are mean ± SEM, n = 6 mice per group (∗∗∗p < 0.001, two-way ANOVA). Representative photographs of tumors in each group are shown (scale bar, 50 mm; n = 6). (D) Experimental lung metastasis of mice inoculated intravenously with B16-EV or B16-sCTLA-4 tumors. Lung tumor nodule frequency and size was measured using stereomicroscopy followed by H&E staining on day 26, with representative samples shown (scale bar, 1 mm). Quantification data are mean ± SD, n = 6 mice per group (∗∗p < 0.01, two-tailed Student’s t test). (E) Immunoblot showing sCTLA-4 in cell-culture supernatant from MCA-205-EV versus MCA-205-sCTLA-4 cells. (F and G) *In vivo* growth of MCA-205 tumors in (F) immunocompetent and (G) immunocompromised NSG mice, n = 7 mice per group (∗∗p < 0.01, two-way ANOVA). (H) Representative immunohistochemistry staining of MCA-205 tumors from (F) with anti-HA (scale bar, 0.5 mm). Data are representative of at least two independent experiments.

To confirm the immunosuppressive effects of sCTLA-4 in an immune-competent host, we monitored growth of murine syngeneic tumor cells constitutively expressing sCTLA-4 *in vivo*. We used B16F10 cells as a model of melanoma, creating cell lines expressing sCTLA-4 (B16F10-sCTLA-4) or empty vector (B16F10-EV). Both cell lines exhibited identical growth profiles *in vitro* (Figures 2A and 2B), but when they were transplanted into mice, B16F10-sCTLA-4 cells showed significantly accelerated growth compared to control (Figure 2C). Similar results were observed in an experimental metastasis model in which B16F10-sCTLA-4 cells spread into the lungs more efficiently and with higher burden than B16F10-EV control cells (Figure 2D). In an alternative fibrosarcoma model in which MCA-205 cells were modified to express sCTLA-4 (MCA-205-sCTLA-4), accelerated malignant cell growth resulting in an approximately 2-fold higher final tumor burden was observed (Figures 2E and 2F). Importantly, transplantation of either MCA-205-sCTLA-4 or MCA-205-EV cells into severely immunocompromised NOD-SCID gamma (NSG) mice revealed overlapping tumor growth profiles (Figures 2G and 2H), indicating that the fitness advantage of MCA-205-sCTLA-4 tumors in syngeneic mice is not intrinsic to the cancer cells but is dependent on extrinsic interaction with an intact immune mechanism.

### sCTLA-4 diminishes tumor-infiltrating T cell activation and function

**Figure 3:**
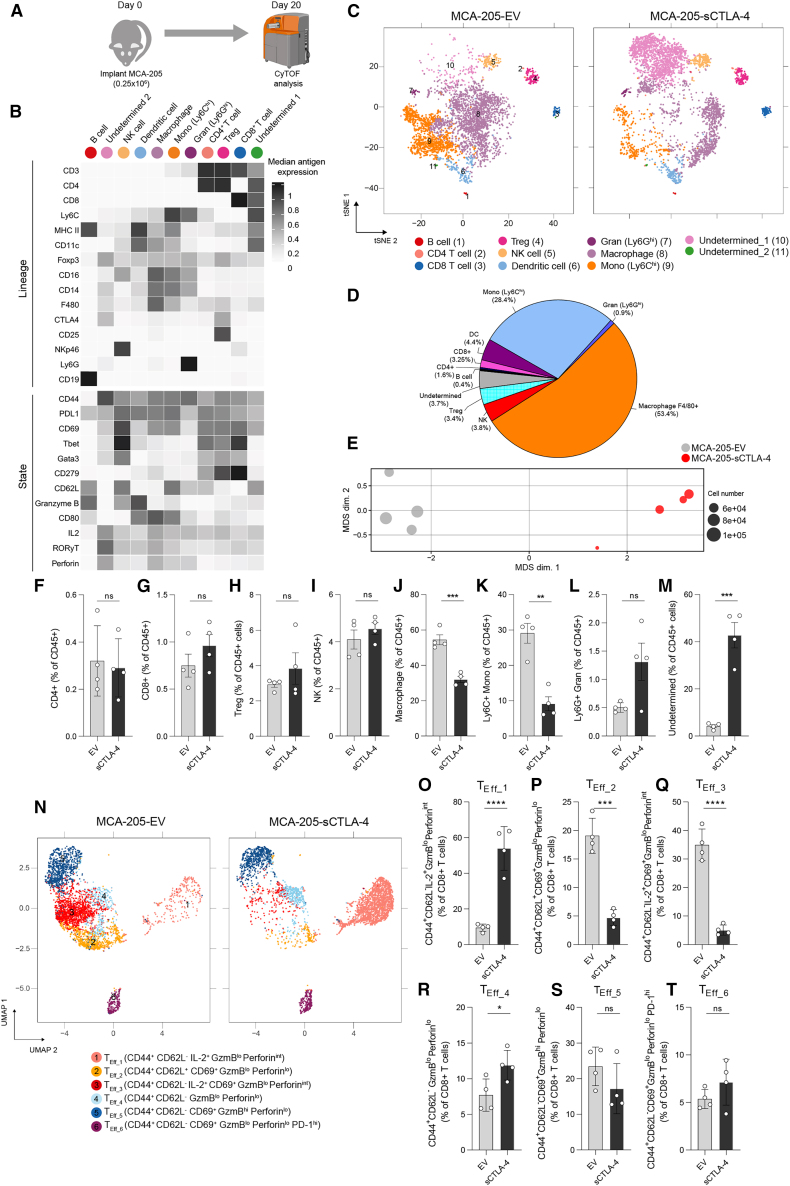
sCTLA-4 inhibits intratumoral T cell activation and differentiation (A) Experimental design for mass-cytometric profiling of MCA-205 tumors on day 20 post inoculation. (B) Heatmap showing the median marker intensity of the 15 lineage markers used for FlowSOM clustering of tumor infiltrates, in addition to functional state marker expression in each cluster. (C) t-SNE analysis of the MCA-205 infiltrates. Twenty-five FlowSOM-identified metaclusters were manually merged according to lineage marker expression. Cells were proportionally combined from EV and sCTLA-4 expressing MCA-205 tumors (n = 7 mice per group) to create the t-SNE plot (1,000 cells per plot for visualization). (D) Relative proportion of each FlowSOM-derived metacluster within MCA-205-EV tumors. (E) Multi-dimensional scaling (MDS) analysis of median antigen expression in MCA-205 tumors; dot size corresponds to the total live CD45^+^ cells obtained for each sample. (F–M) Comparison of the proportion of the indicated cell populations within tumor infiltrates. (N) Uniform manifold approximation and projection (UMAP) of CD8^+^ T cell subsets. CD8^+^ T cells identified in (B) were reclustered on functional state marker expression. The six clusters identified by FlowSOM were manually annotated as: T_Eff_1_ (CD44^+^ CD62L^−^ IL-2^+^ granzyme B^lo^ perforin^int^); T_Eff_2_ (CD44^+^ CD62L^+^ CD69^+^ granzyme B^lo^ perforin^lo^); T_Eff_3_ (CD44^+^ CD62L^−^ IL-2^+^ CD69^+^ granzyme B^lo^ perforin^int^); T_Eff_4_ (CD44^+^ CD62L^−^ granzyme B^lo^ perforin^lo^); T_Eff_5_ (CD44^+^ CD62L^−^ CD69^+^ granzyme B^hi^ perforin^lo^); and T_Eff_6_ (CD44^+^ CD62L^−^ CD69^+^ granzyme B^lo^ perforin^lo^ PD-1^hi^). (O–T) Quantification of CD8^+^ T cell subset frequency. Data in (F)–(M) and (O)–(T) are expressed as mean ± SD; n *=* 4 mice per group. Statistical significance was calculated using two-tailed Student’s t test (∗∗∗p *<* 0.0001; ∗∗∗p *<* 0.001; ∗p *<* 0.05; ns [not significant], p > 0.05). Data are representative of two independent experiments. GzmB, granzyme B.

The accelerated growth kinetics of sCTLA-4-secreting tumors was further investigated within the MCA-205 fibrosarcoma model. Here, we focused on profiling the immune infiltrate using mass cytometry, comparing mice exposed to MCA-205-sCTLA-4 and MCA-205-EV cells 20 days after implantation (Figure 3A). Unsupervised clustering using flow self-organizing maps (FlowSOM) was performed on data derived from live CD45^+^ cells within tumor infiltrates. This approach identified 11 cell populations, of which nine had characteristics that allowed assignment to distinct immune cell subsets while two had characteristics which, although myeloid in nature, remained undetermined based on current understanding of myeloid cell phenotypes (Figure 3B). These clusters were then visualized within t-distributed stochastic neighbor embedding (t-SNE) projections of the data to assess the relative proportions of each population of cells (Figures 3C and 3D).

Overall, myeloid subpopulations such as monocytes, dendritic cells (DCs), and macrophages represented the majority of the immune component of the tumor microenvironment (TME) (∼30% and ∼60% of CD45^+^ cells were monocytes and macrophages, respectively). Importantly, t-SNE projection of the data showed distinct changes to each subpopulation of cells associated with cell infiltrates from MCA-205-sCTLA-4 and MCA-205-EV tumors (Figure 3C), an observation that was confirmed by multi-dimensional scaling analysis of median antigen expression values associated with all CD45^+^ cells from each model (Figure 3E). Comparison of the immune cell composition within MCA-205-sCTLA-4 and MCA-205-EV tumors (Figures 3F–3M) revealed a dramatic reorganization of the myeloid compartment, with reduced frequencies of both F4/80^+^ macrophages and Ly6C^hi^ monocytes in MCA-205-sCTLA-4 tumors (Figures 3J and 3K). In contrast, within the overall lymphoid compartment, there were no differences in frequency of B and NK cells, or of CD4^+^, CD8^+^ T cells, and T_reg_ cells between MCA-205-sCTLA-4 tumors and EV controls (Figures 3F–3I). Importantly, the ratio of CD8^+^ to T_reg_ cells showed no change (Figure S5A). The reduction of macrophages and monocytes occurred alongside a significant enrichment of an undetermined population of CD45^+^ cells characterized by expression of F4/80 and FoxP3 within infiltrates from MCA-205-sCTLA-4 tumors (Figure 3M). This undetermined population of CD45^+^ cells retains myeloid features and is similar in phenotype to a subpopulation of F4/80^+^/FoxP3^+^ macrophages reported to infiltrate lesions resulting from ischemic stroke.^29^ This report shows that such FoxP3^+^ macrophages have enhanced ability to scavenge debris and so may have a similar role within the tumors of our model.

Our previous *in vitro* experiments showed that sCTLA-4 suppresses T cell activation, and we next investigated this within the population of tumor-infiltrating T lymphocytes (TILs) in our system. To this end, we examined CD8^+^ T cell phenotypes by reclustering according to functional state marker expression^30^ (Figures 3N, S5B, and S5C). We identified six cell subsets, all of which exhibited CD44 expression but had variable expression of functional markers. We observed a marked enrichment of cells in the T_Eff_1_ subset within MCA-205-sCTLA-4 tumors, characterized by an absence of CD62L and moderate interleukin-2 (IL-2) and perforin expression (Figure 3O). This was accompanied by a significant reduction in the frequency of the large T_Eff_2_ and T_Eff_3_ subsets, both characterized by expression of the activation marker CD69 (Figures 3P and 3Q). The T_Eff_4_ subset, distinguished by a lack of activation markers and low cytolytic protein expression, was also enriched in sCTLA-4-expressing tumors (Figure 3R). The T_Eff_5_ subset exhibiting high granzyme B expression was less abundant in the presence of sCTLA-4, although this did not reach statistical significance (Figure 3S). Cells within the T_Eff_6_ subset, exhibiting high programmed cell death protein 1 (PD-1) and CD69 expression, potentially reflecting an exhausted or terminally differentiated phenotype, were present at similar frequencies between the tumor types (Figure 3T). These results therefore confirm our *in vitro* data and show that secretion of sCTLA-4 by tumor cells actively suppresses T cell activation *in vivo*. Collectively, these data suggest that sCTLA-4 profoundly modifies the immune context of the TME and dampens T cell activation and effector function.

### Isoform-specific sCTLA-4 antibody augments anti-tumor immune responses

**Figure 4:**
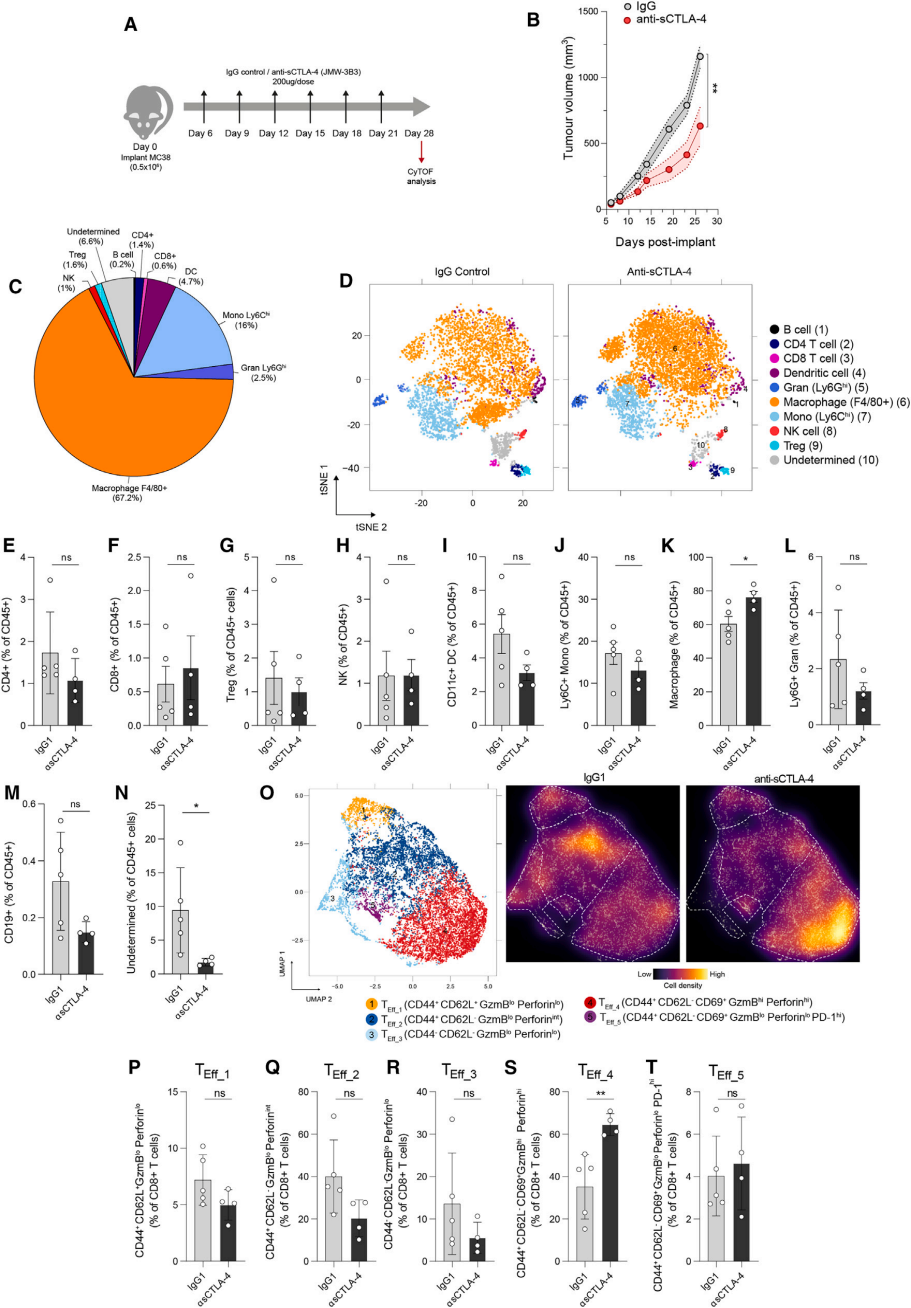
sCTLA-4 blockade promotes T cell cytotoxic function and attenuates murine tumor growth *in vivo* (A) Schematic showing study design and schedule for the treatment of mice bearing MC38 tumors. (B) *In vivo* growth of tumors treated according to (A). Data are mean ± SEM, n = 16–18 mice per group, from three independent experiments (∗∗p < 0.01, two-way ANOVA). (C) Proportions of major tumor-infiltrating leukocyte populations within control MC38, expressed as percentage of CD45^+^ cells. (D) t-SNE analysis of immune infiltrates isolated from MC38 tumors in (B) at day 28. Cell lineage markers were used for FlowSOM-based metaclustering of CD45^+^ cells. Twenty-five FlowSOM-identified metaclusters were manually merged according to lineage marker expression. Clustering was performed on all cells from both treatment groups (n = 4–6 mice per group), with 1,000 cells visualized in t-SNE plots. (E–N) Comparison of proportions of the indicated cell populations between anti-sCTLA-4- or isotype control-treated tumors. (O) UMAP of CD8^+^ T cell subsets. CD8^+^ cells identified in (D) were reclustered using functional state marker expression. Five clusters identified by FlowSOM were manually annotated as: T_Eff_1_ (CD44^+^ CD62L^+^ granzyme B^lo^ perforin^lo^); T_Eff_2_ (CD44^+^ CD62L^−^ granzyme B^lo^ perforin^int^); T_Eff_3_ (CD44^-^ CD62L^−^ granzyme B^lo^ perforin^lo^); T_Eff_4_ (CD44^+^ CD62L^−^ CD69^+^ granzyme B^hi^ perforin^hi^); and T_Eff_5_ (CD44^+^ CD62L^−^ CD69^+^ granzyme B^lo^ perforin^lo^ PD-1^hi^). Shifts in CD8 functionality following treatment are visualized by cell density scaling on the CD8^+^ T cell subset UMAP. (P–T) Quantification of CD8^+^ T cell subset frequency in treated tumors. Data in (E)–(N) and (P)–(T) are expressed as mean ± SD; n = 4–5 in each group. Statistical significance was calculated using two-tailed Student’s t test (∗∗p *<* 0.01; ∗p *<* 0.05; ns [not significant], p > 0.05). Data are representative of two independent experiments. GzmB, granzyme B.

Having established that the presence of sCTLA-4 in the TME suppresses intratumoral T cell activation and effector functionality *in vivo*, we sought to determine the effect of antibody-mediated sCTLA-4 blockade on immune cell profiles and T cell responses within a transplantable syngeneic tumor. Genetic ablation of CTLA-4 in mice has been shown to result in multi-organ toxicity and lethal phenotype,^31^^,^^32^ while administration of pan-CTLA-4 antibody to naive mice can lead to spontaneous development of autoimmune diseases.^33^ To ensure that sCTLA-4 blockade did not induce such gross immune defects in normal immunocompetent hosts, we treated naive C57BL/6 mice with anti-sCTLA-4 antibody twice a week for 5 weeks and assessed immune cell architecture in the primary and secondary lymphoid organs (thymus, spleen, and lymph nodes) (Figure S6). We detected no changes in the immune cell composition of these organs, providing assurance that administration of isoform-specific antibody does not disrupt overall immune homeostasis in naive mice. We then treated mice transplanted with syngeneic MC38 colorectal tumors with anti-sCTLA-4 or isotype control antibody every 3 days until day 21 (Figure 4A). Anti-sCTLA-4 antibody treatment significantly attenuated tumor growth rate, inhibiting tumor growth by approximately 50% relative to control-treated mice (Figure 4B). As MC38 themselves are unlikely to be the source of sCTLA-4 (data not shown), immune infiltrates from the tumors were subjected to mass cytometry analysis at day 28 post inoculation to elucidate the cellular changes underlying this anti-tumor efficacy. Myeloid cell populations, including F4/80^+^ macrophages and Ly6C^hi^ monocytes, represented the major component of MC38 immune infiltrates (∼85%) (Figures 4C, 4D, and S7A) and were largely unaffected by treatment with anti-sCTLA-4 (Figures 4I–4L). With respect to lymphoid cells, infiltrates of anti-sCTLA-4-treated tumors exhibited comparable frequencies of CD8^+^, CD4^+^ T cells, T_reg_ cells, and NK cells compared to control-treated tumors (Figures 4E–4H), with conservation of T cell to T_reg_ cell ratios between the treatment groups (Figure S7B). B lymphocytes seemed less abundant in anti-sCTLA-4 treated tumors relative to controls (Figure 4M), and there was clear reduction of an undetermined population characterized by F4/80 and FoxP3 expression within the infiltrates from anti-sCTLA-4 treated mice (Figure 4N). To assess the effect of sCTLA-4 inhibition on T cell function, CD8^+^ T cells were reclustered according to state marker expression (Figures 4O, S7C, and S7D). We identified five cell subsets with variable expression of functional markers, with clusters distinct from those found within MCA-205 tumors characterized above. Cells within the T_Eff_1_ subset exhibited both CD44 and CD62L expression with a lack of activation and cytolytic markers. Although reduced in treated tumors, this did not reach statistical significance (Figure 4P). Cells within T_Eff_2_ were distinguished from T_Eff_1_ by an absence of CD62L and gain of low-level perforin expression. While also exhibiting reductions in treated tumors, this did not reach statistical significance (Figure 4Q). Cells within the T_Eff_3_ subset exhibited a lack of CD44, CD62L, and activation marker expression and were largely unchanged between control and treated tumors (Figure 4R). Notably, we observed a significant enrichment of T_Eff_4_ cells, characterized by CD69 and high-level granzyme B expression, within tumors treated with anti-sCTLA-4 (Figure 4S), indicating that sCTLA-4 blockade promotes the activation and differentiation of TILs. Indeed, this was the most abundant subset in treated tumors. The last identified subset, T_Eff_5,_ exhibited a lack of cytolytic marker expression but marked PD-1 positivity and was unchanged by treatment with anti-sCTLA-4 (Figure 4T). Interestingly, when we repeated the therapy model using 4-fold higher inoculum of MC38 tumors (2 × 10^6^ cells), we observed loss of therapeutic efficacy of anti-sCTLA-4, thereby achieving similar dynamics of growth between immunoglobulin-G- and anti-sCTLA-4-treated groups, which also resulted in loss of enrichment of the T_Eff_4_ cluster of activated T cells (Figure S8). This suggests that when therapeutic efficacy is achieved, the enhanced, anti-tumoral CD8^+^ T cell response is driven by anti-sCTLA-4.

## Discussion

Most studies do not consider the possible immunoregulatory impact of the soluble form of CTLA-4 even though it is evident that blocking CTLA-4 can trigger anti-tumor immunity by promoting the infiltration of cytotoxic effector T cells into tumors.^34^ Currently used immune checkpoint inhibitor antibodies, such as ipilimumab, have revolutionized cancer therapy, but the advantages of these antibodies are restricted to a small subset of patients and are coupled with toxicities and side effects of varying severity, which can sometimes be so intense that patients may have to discontinue treatment and receive immunosuppressive therapy instead.^35^ Understanding isoform-specific biology and function of immune checkpoint proteins provides an opportunity to optimize cancer immunotherapy by taking into account the relative roles of each isoform.^22^ Dissection of the division of labor between CTLA-4 isoforms to processes such as tumor tolerance and immune evasion may reveal divergent functions which may result in reduced toxicity and should be considered for more effective cancer therapy.

Despite growing clinical applications of anti-CTLA-4 antibodies for the treatment of cancer, its precise mechanisms of action remain poorly defined. The current perception of CTLA-4 biology focuses almost entirely on the membrane-bound receptor, and it is the activity of this isoform that almost all studies focus on to elicit greater anti-tumor immune responses. As a result, there has been a tendency to assume that checkpoint regulation operates solely through membrane-based interactions, dismissing any contribution of the soluble isoform in this process. However, our data provide direct evidence that sCTLA-4 mediates modulation of murine and human T cell activation in a range of situations, exhibiting function similar to that of the engineered soluble dimeric fusion protein CTLA4-Fc. Our analysis of CTLA-4 mRNA level within human tumor samples showed significant positive correlations between isoforms, and membrane-bound CTLA-4 is the predominant isoform. Although the CTLA-4 isoforms are mostly known to be T cell derived, many human cancer cell types express both CTLA-4 transcripts and functional CTLA-4, detectable in the cytoplasm or on the cell surface.^36^ Evaluating the relative levels of sCTLA-4:CTLA-4 in tumor types may be important in the context of CTLA-4-based immune checkpoint therapy. Given the difficulty in manipulating primary cells and lack of clear insight at present as to the most appropriate cell to target, we used a system in which sCTLA-4 is overexpressed by the tumor to study its effects on the TME. This has enabled us to demonstrate for the first time that sCTLA-4 promotes tumor growth and modifies intratumoral CD8^+^ T cell phenotype. In sCTLA-4-expressing tumors, we observed a significant enrichment of the T_Eff_1_ subset characterized by intermediate to low-level perforin expression. Interestingly, there were no CD8^+^ subsets exhibiting high expression of both perforin and granzyme B, although T_Eff_5_ clearly expressed high levels of granzyme B and the data trend toward a depletion of this cluster within MCA-205-sCTLA-4 tumors. The dual high-level expression of CD44 and CD62L in T_Eff_2_ potentially indicates a central memory phenotype, a cell type that typically lacks effector function but has high proliferative capacity.^37^ Similarly, IL-2 expression and CD69 positivity in T_Eff_3_ indicate that these cells are activated and are producing a cytokine important in stimulating T cell proliferation and effector differentiation. A higher frequency of T_Eff_2_ and T_Eff_3_ cells in EV tumors may promote the generation of sufficient effector T cells to control tumor growth. Thus, enrichment of T_Eff_1_ and loss of T_Eff_2-3_ may in part account for the accelerated tumor growth due to sCTLA-4. Conversely, during analysis of CD8^+^ T cells within MC38 tumors treated with anti-sCTLA-4, we observed an enrichment of a highly cytotoxic cluster, T_Eff_4_, which was characterized by high-level dual granzyme B and perforin expression. Significantly, neither enrichment of T_Eff_4_ nor therapeutic efficacy of anti-sCTLA-4 was observed following inoculation of higher tumor cell numbers, highlighting the potential functional significance of this subset in tumor clearance.

sCTLA-4 expression is not confined to T cells and may also have roles in regulating processes involved in myeloid cell biology. Indeed, in our preclinical studies we found an unexpected and dramatic remodeling of the myeloid compartment following sCTLA-4 manipulation. Although it is difficult to discern whether these changes are direct or indirect as a result of the changes to the T cells, myeloid cell types are renowned for high functional plasticity, and expression of both CTLA-4 isoforms has been reported.^38^^,^^39^ However, their function in myeloid cells is a much less well studied aspect of CTLA-4 biology.^28^ Specifically, previous reports of DC-secreted CTLA-4 have shown downregulation of bystander B7 ligand availability and downstream negative effects on CD8^+^ T cell responses.^38^ Owing to the simultaneous detection of 25 or more cellular markers, mass-cytometric analysis and unsupervised clustering can often reveal unconventional and unknown populations. Using this approach, we observed an unconventional myeloid population consistently enriched or depleted following sCTLA-4 overexpression or blockade, respectively, which may represent a functionally significant population in the TME.

Some human cancer cells have been found to produce sCTLA-4 naturally,^15^^,^^40^^,^^41^ and we predict that this may lead to blunted T cell effector activity and immune escape. This is further supported by the finding that cancer cell-intrinsic expression of sCTLA-4 did not provide growth advantage to cancer cells *in vitro* or in an immunocompromised host, evidence that largely favors the hypothesis that immune cells, particularly CD8^+^ T cells, are being held at bay by sCTLA-4. Additionally, in the absence of sCTLA-4 secretion by cancer cells, immune cell populations including T_reg_ cells are known to produce sCTLA-4 and curtail anti-tumor immune responses.^23^^,^^42^ Conventional anti-CTLA-4 antibodies such as ipilimumab are likely to block both membrane and soluble isoforms, as they are targeted against an epitope in the extracellular region that is present in both isoforms.^11^^,^^43^ Given the immunological effects achieved by isoform-specific targeting of sCTLA-4, it is feasible that the anti-tumor activity of conventional pan-CTLA-4 antibodies occurs at least in part through blockade of sCTLA-4. Acknowledging the relative role of sCTLA-4 in health and disease may lead to distinct interpretation of many observations concerning membrane-bound CTLA-4 function, relevant across an enormous range of clinical applications including cancer, autoimmunity, allergy, and transplant biology.

## Materials and methods

### Cell lines

Mouse tumor cell lines MC38 and B16F10 were obtained from the ATCC (London, UK) while MCA-205 were from Sigma-Aldrich. Human HEK 293T cells were obtained from ATCC. B16F10 melanoma cells were cultured in DMEM containing 10% fetal bovine serum (FBS). MC38 cells were grown in DMEM with 10% FBS, 2 mM glutamine, 0.1 mM non-essential amino acids, 1 mM sodium pyruvate, 10 mM HEPES, 50 μg/mL gentamycin sulfate, penicillin/streptomycin, and MCA-205 in RPMI-1640 containing 2 mM L-glutamine, 1 mM sodium pyruvate, 10% FBS, and 0.1 mM non-essential amino acids (Sigma/Gibco) at 37°C and 5% CO_2_. All cell lines were routinely tested for mycoplasma infection by PCR. Human PBMCs were purified from healthy donor leukocyte cones purchased from Blood and Transplant (Liverpool, UK) by Lymphoprep (Serumwerk) density centrifugation and cultured in RPMI-1640 + 10% fetal calf serum (FCS)/1% penicillin/streptomycin.

### Mice

All animal studies were performed under UK Home Office Project License PP6634992, in accordance with the UK Animal (Scientific Procedures) Act 1986 and the EU Directive 86/809. Studies were approved by the University of Liverpool Animal Welfare and Ethical Review Body and referred to the Workman guidelines.^44^

### Preclinical tumor models

Tumor cells were harvested in log-phase growth by detachment with 0.25% trypsin-EDTA (Gibco) solution and washing plus resuspension in PBS. For *in vivo* growth studies, 5 × 10^5^ B16F10 or MC38 or 2 × 10^5^ MCA-205 were injected subcutaneously into the right flank of female C57BL/6J or NSG mice (6–8 weeks of age). Tumor growth was measured using calipers and the volume formula (width^2^ × length)/2, with a tumor burden limit of 1,500 mm^3^. For experimental metastasis models measuring B16F10 lung engraftment, 2 × 10^5^ cells were injected into the tail vein of 6- to 8-week-old female C57BL/6 mice.

### Immunoblot

Cell extracts were prepared using RIPA buffer (Thermo Fisher Scientific), with extracts or cell-culture supernatant being diluted in sample buffer with β-mercaptoethanol. Information on antibodies used is given in Table S1. After SDS-PAGE, proteins were transferred onto polyvinylidene fluoride membranes (Merck) and blocked using 5% non-fat dried milk in PBS-Tween 20. Following overnight incubation in primary antibody, membranes were washed and incubated in horseradish peroxidase (HRP)-conjugated secondary antibody before detection using Immobilon ECL Ultra substrate (Millipore). For mobility shift assays, samples were incubated with PNGase F (NEB) to deglycosylate *N*-linked glycans.

### Flow cytometry

PBMCs were washed once in FACS buffer (2% FBS in PBS) before being stained with antibodies (Table S1) in FACS buffer (1:100 v/v) and incubated on ice for 45 min. Samples were then washed twice by centrifugation at 500 × *g* for 5 min and resuspension in FACS buffer. For measurement of CTLA-4 in adherent tumor cells, cells were collected after incubation in trypsin-EDTA solution (Gibco). HeLa and B16F10 were then stained with anti-human-CTLA-4-APC (BNI3, BioLegend) and anti-mouse-CTLA-4-APC (UC10-4B9, BioLegend), respectively. For staining of intracellular protein, samples were fixed in 4% paraformaldehyde for 15 min at room temperature before permeabilization with the eBioscience Foxp3/Transcription Factor Staining Buffer Set (Thermo), according to the manufacturer’s instructions. All samples were analyzed using an Attune NxT flow cytometer (Invitrogen), with .fcs files being analyzed with FlowJo v10 (Tree Star).

### *In vitro* co-cultures

Human PBMCs were stained with 5 μM CellTracker carboxyfluorescein diacetate succinimidyl ester (CFSE) (Invitrogen). PBMCs and HeLa target cells were co-cultured in U-bottom 96-well plates at a range of target-effector ratios (indicated in figure legends). T cells were stimulated with 10 μg/mL plate-immobilized anti-CD3 (OKT3, BioLegend) and cultured for 4 days prior to staining with anti-CD8-APC (SK1, BioLegend) and proliferation measured by flow cytometry, judged by CFSE dilution. For T cell killing assays, following PBMC-HeLa co-cultures in 12-well plates, suspension cells were removed by PBS washes. HeLa cells which remained viable and adherent were then fixed in absolute methanol (Sigma-Aldrich) for 20 min at room temperature before staining with 0.5% crystal violet solution (Sigma-Aldrich). For transwell co-cultures, 2.5 × 10^4^ HeLa cells were seeded into the lower compartment of a 24-well plate, with 1 × 10^5^ PBMCs seeded into the upper 0.4-μm ThinCert cell-culture insert (Greiner Bio-One, Austria). Cells were cultured for 4 days in RPMI-1640 medium with 10% FBS + 1% penicillin/streptomycin supplemented with 2 μg/mL anti-CD3 to activate PBMCs (OKT3, BioLegend). CD8^+^ T cell proliferation was measured by following the same method described for co-cultures above. NK cells were depleted by positive selection using the EasySep Human CD56 Positive Selection Kit II (STEMCELL Technologies).

### *In vitro* T cell suppression assay

To measure the suppression of murine T cell proliferation by sCTLA-4, 1 × 10^5^ CFSE-stained splenocytes were co-cultured with bone marrow-derived macrophages (BMDMs) (20:1 splenocyte to BMDM) in 96-well U-bottom plates with RPMI-1640 + 10% FCS + 0.05 mM β-mercaptoethanol. Cultures were stimulated with PMA (5 ng/mL, Alfa-Aesar) + ionomycin (0.5 μg/mL, Thermo Fisher Scientific) and supplemented with sCTLA-4 conditioned medium or murine CTLA-4-Fc fusion protein (10 μg/mL, BioLegend). After 4 days, cells were harvested and stained with anti-mouse-CD8α (BioLegend) for analysis of T cell proliferation by flow cytometry as described above.

### BMDM isolation and culture

Femurs and tibias from 6- to 8-week-old female C57BL/6J mice were flushed with ice-cold PBS into Petri dishes. The suspension was then pressed through 70-μm cell strainers (Corning) before centrifugation at 200 × *g* for 5 min at 4°C. Bone marrow cells were differentiated into BMDMs by culturing them in DMEM + 10% FCS + 25 ng/mL recombinant murine macrophage colony-stimulating factor (BioLegend) at a density of 1 × 10^6^ cells/mL for 7 days, with the culture medium being replaced every 2 days.

### Cloning

Expression plasmids were constructed for the stable overexpression of human and mouse sCTLA-4 in various tumor cell lines. Human and murine sCTLA-4 open reading frames (ORFs) were PCR-amplified from the cDNA of anti-CD3 activated PBMCs or splenocytes, respectively. Amplification of ORFs used the following primer sequences: 5′-ATG GCT TGC CTT GGA TTT CAG-3′ (human sCTLA-4 forward), 5′-AGT CAC ATT CTG GCT CTG TTG G-3′ (human sCTLA-4 reverse), 5′-ATG GCT TGT CTT GGA CTC CG-3′ (murine sCTLA-4 forward), and 5′-TCA CAT TCT GGC TCT GTT GG-3′ (murine sCTLA-4 reverse). The sCTLA-4 ORF was cloned into a pCDH-EF1-FHC lentiviral expression vector by restriction digest and ligation with T4 DNA ligase (Thermo Fisher Scientific) to produce pCDH-EF1-sCTLA-4 expression vectors. pCDH-EF1-FHC was a gift from Richard Wood at MD Anderson Cancer Center, University of Texas (Addgene plasmid #64874).

### Generation of stable expression cell lines

For lentivirus generation, 4 × 10^5^ HEK 293T cells were transfected with pCDH-EF1-sCTLA-4 expression plasmid (1.5 μg) and psPax2 (2 μg) and pMD2.G (1.5 μg) , using Viafect (Promega) and dilution in OptiMEM (Gibco). Packaging and envelope plasmids were gifts from Didier Trono (Addgene plasmid #12260 and #12259, respectively). Medium was replaced on the following morning with fresh DMEM + 10% FCS. Lentiviral supernatant was collected 48–72 h post transfection, clarified by centrifugation at 500 × *g* for 5 min, and filtered using 0.45-μm polyethersulfone filters (Starlab). For lentiviral transduction, 2 × 10^5^ target tumor cells were seeded in 6-well plates and infected with lentivirus + polybrene (8 μg/mL) before selection of transduced cells using 4 μg/mL puromycin (Sigma-Aldrich).

### Isolation of tumor-infiltrating leukocytes

Excised tumors were mechanically disrupted and incubated with 1.67 Wünsch U/mL Liberase TL (Roche) and 0.2 mg/mL DNase I (Merck) for 30 min at 37°C with agitation. Digested tumors were homogenized by pipetting before being passed through 100-μm nylon mesh strainers (Corning). Cell suspensions were washed with RPMI-1640 before proceeding to mass cytometry staining.

### Mass-cytometric immunophenotyping

Custom antibody-metal conjugations were prepared using the Maxpar Antibody Labeling Kit (Standard BioTools) according to the supplied protocol recommendations. Thereafter, Maxpar-conjugated antibodies were stored in PBS-based antibody stabilization solution (Candor Biosciences) at 4°C and titrated before use. Isolated TILs were washed with PBS prior to viability staining with Cell-ID cisplatin (Standard BioTools). Samples were then barcoded using metal-labeled anti-mouse CD45, and an equal number of cells from each sample was pooled for subsequent staining. Samples were incubated with TruStain FcX (anti-mouse CD16/32) for 10 min on ice to block Fc receptors before immunostaining with antibodies for lineage and state-defining markers (Table S2) for 45 min on ice. Samples were then washed with cell-staining buffer (Standard BioTools) before fixation in 4% paraformaldehyde (Thermo Fisher Scientific) for 15 min at room temperature. For measurement of intracellular cytokines, samples were processed using the FoxP3/Transcription Factor Fix-Perm Kit (eBioscience) before staining for 1 h at room temperature. After washing in cell staining buffer, samples were incubated with Cell-ID Intercalator-Ir (Standard BioTools) for doublet discrimination prior to analysis. Data were preprocessed to isolate live CD45^+^ cells using FlowJo (BD) before analysis, and unsupervised clustering was performed using the R-based package CATALYST.^45^

### Immunohistochemistry

Tumors were excised and fixed in 10% neutral-buffered formalin (Sigma-Aldrich) for 48 h at 4°C with rotation. Deparaffinization and antigen retrieval on sections was performed using the Dako PT-link station (Agilent) before immunostaining overnight at 4°C with anti-HA (CST) antibody diluted 1:1,000 in blocking solution (3% BSA/PBS). For secondary staining, the HRP-labeled polymer from the EnVision+ system was used (Dako) according to supplied instructions. Staining was developed using diaminobenzidine and counterstained with hematoxylin (Sigma-Aldrich).

### RNA-seq patient datasets

Normalized isoform-level RNA-seq data were obtained through The Cancer Genome Atlas Data Matrix portal (level 3, tcga-data.nci.nih.gov/tcga/dataAccessMatrix.htm) and Firebrowse (firebrowse.org/). Transcript isoform identification codes were mapped to gene names using the UCSC table browser.^25^

### Statistical analysis

Data are expressed as means ± SEM unless otherwise indicated. Student’s t test and two-way ANOVA were performed for the statistical analysis, the details of which can be found in the figure legends. To measure differences in mean tumor size between mice implanted with tumor cell lines at multiple time points throughout the study, two-way ANOVA was used followed by Sidak’s post hoc test to correct for multiple comparisons. Use of two-way ANOVA assumes no missing values at the time point being analyzed. Since we were measuring the effect of the cell line on growth only, we applied a main-effects model which handles missing values assuming they appear at random. Missing values in these studies are caused by early euthanasia of mice due to humane endpoints, namely tumor ulceration. Statistical analysis was performed with GraphPad Prism (version 9.5.0, GraphPad Software). p values of less than 0.05 were considered significant.

## Data and code availability

All data generated are presented in the figures and supplemental information. Materials are available upon reasonable request.

## Supplemental information

Document S1. Figures S1–S8 and Tables S1 and S2

Document S2. Article plus supplemental information
