# Supplementary material for: Soluble CTLA-4 attenuates T cell activation and modulates anti-tumor immunity
Source: Mol Ther. 2023 Dec 5;32(2):457–68. doi: 10.1016/j.ymthe.2023.11.028 (PMC10861965; doi:10.1016/j.ymthe.2023.11.028)
Supplement: Document S1. Figures S1–S8 and Tables S1 and S2 [file mmc1.pdf]

YMTHE, Volume *32*

Supplemental Information

Soluble CTLA-4 attenuates T cell activation and modulates anti-tumor immunity

Paul T. Kennedy, Emma L. Saulters, Andrew D. Duckworth, Yeong Jer Lim, John F. Woolley, Joseph R. Slupsky, Mark S. Cragg, Frank J. Ward, and Lekh N. Dahal

Supplemental data

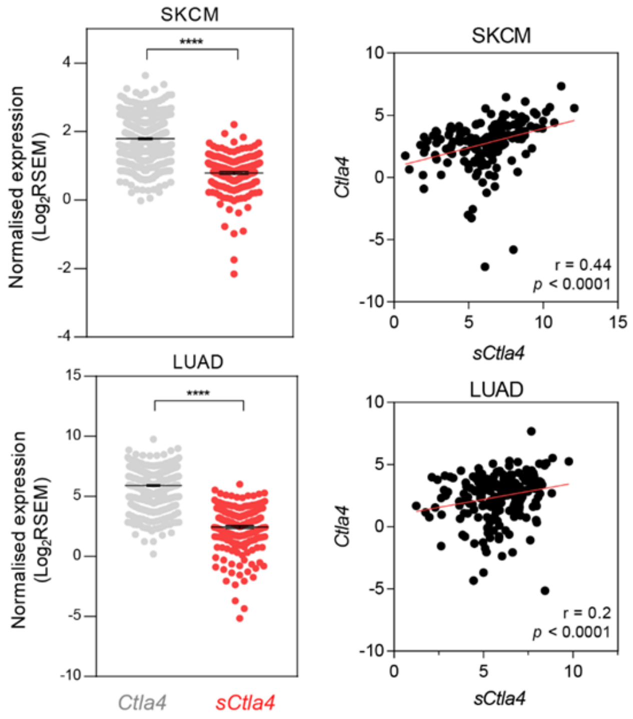

Figure S1. sCTLA-4 expression correlates with membrane CTLA-4 isoform levels. Quantification of soluble and transmembrane *Ctla-4* mRNA levels in SKCM and LUAD TCGA bulk tumour RNAseq datasets (**** p<0.0001 Kolmogorov-Smirnov test) and correlation analysis of bulk tumour soluble and membrane-bound CTLA4 mRNA. Pearson's coefficient is given.

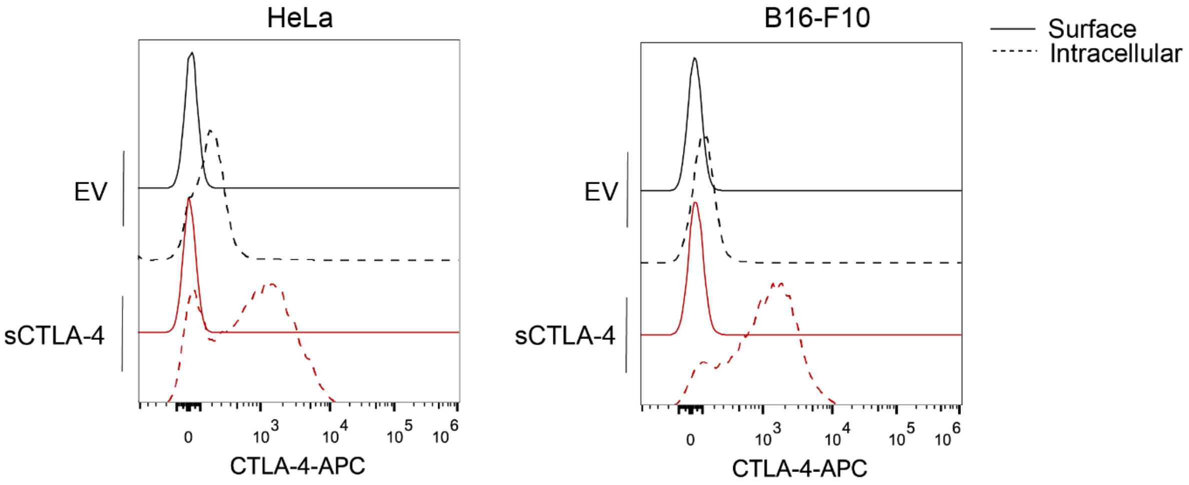

Figure S2. Flow cytometric analysis of surface and intracellular CTLA-4 in cells expressing recombinant sCTLA-4. Histograms show CTLA-4 signal intensity for both surface and intracellular CTLA-4 in HeLa and B16-F10 cells.

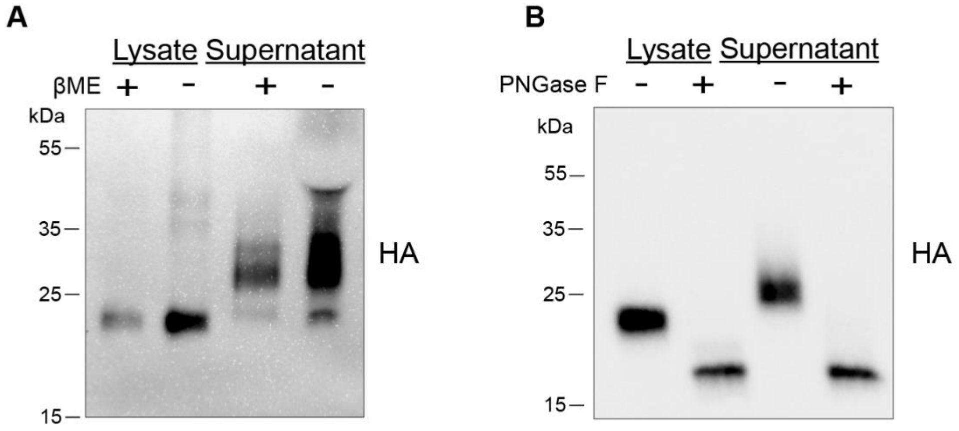

Figure S3. Recombinant sCTLA-4 is secreted as a glycosylated dimer. (A) Immunoblot showing lysates and supernatant derived from HeLa-sCTLA-4 cells. For reducing samples, samples were boiled for 5 minutes in the presence of beta-mercaptoethanol (βME) to reduce disulphide linkages. **(B) sCTLA-4 is glycosylated before secretion.** Cell lysates and supernatant were incubated with PNGase F and then subjected to SDS PAGE and blotting for HA. The data show the secreted supernatant has a higher mobility which than pre-secreted material and normalisation of this after PNGase F treatment indicate that this difference is due to N glycosylation prior to secretion.

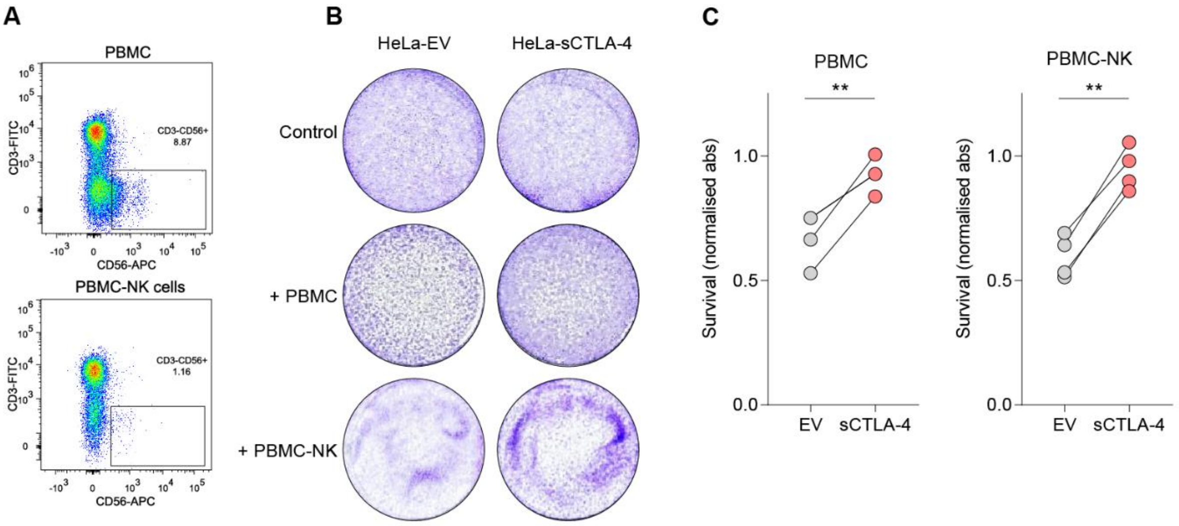

Figure S4. Immune cell-mediated cytotoxicity in HeLa co-cultures is not NK cell dependent. T cell-mediated tumour cell killing assay of HeLa-sCTLA-4 cells. **(A)** Flow cytometry showing depletion of NK cells from PBMCs prior to co-culture with HeLa-sCTLA-4 cells. **(B)** Representative images showing showing crystal violet-stained viable HeLa cells following co-culture with anti-CD3 activated PBMCs or PBMCs-NK cells. **(C)** Quantification of crystal violet absorbance. Data represent 4 independent PBMC donors (**p<0.01 Student's t-test).

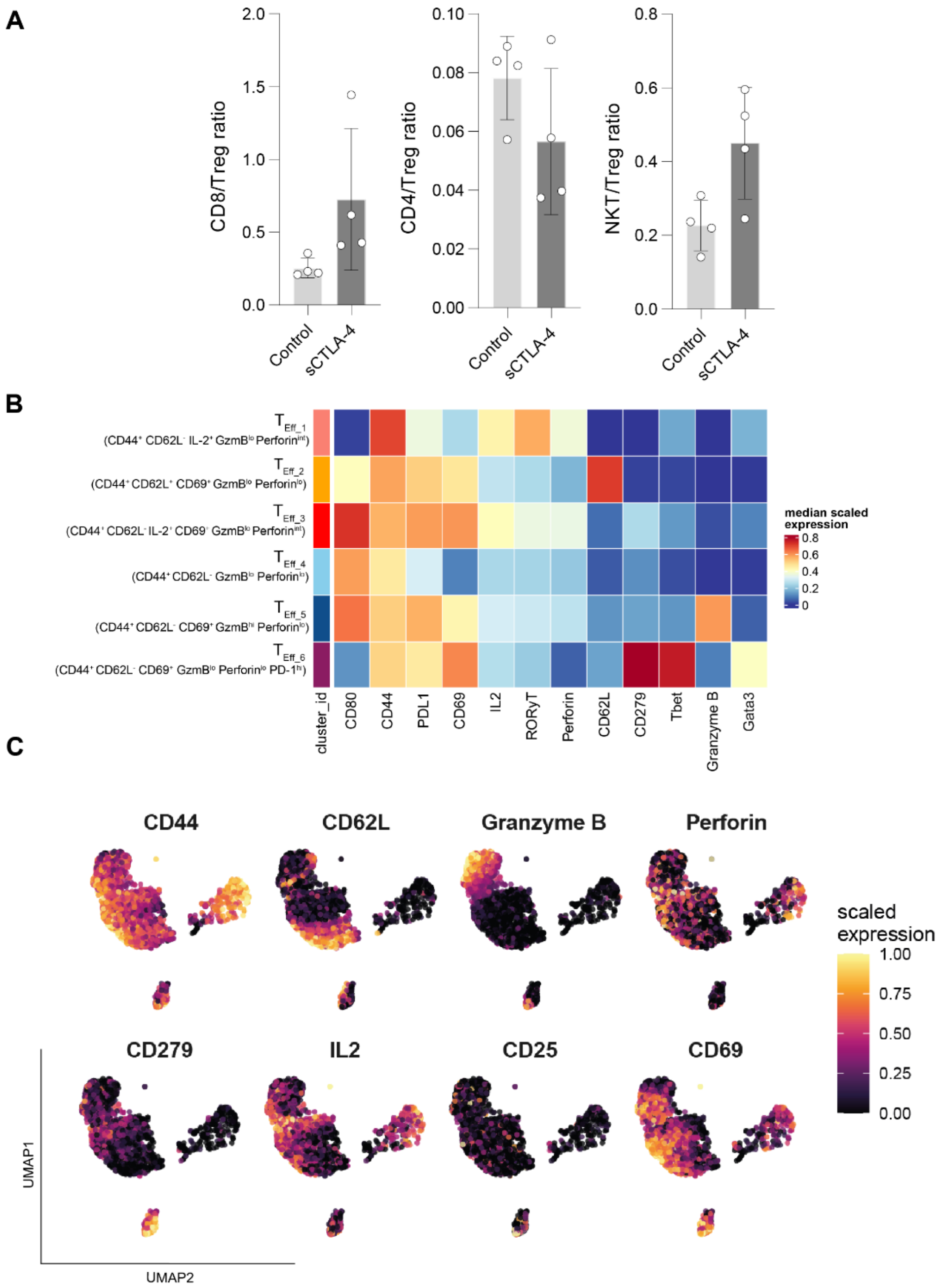

Figure S5. Lymphocyte:Treg ratios and scaled CD8 T-cell marker expression in MCA-205 tumours. (A) Ratio of CD8, CD4 and NKT to Treg in tumour infiltrates bearing MCA-205-EV control or MCA-205-sCTLA-4 tumours. Data are expressed as mean ± SD; n = 4 mice per arm. Statistical significance was calculated using two-tailed Student's t-test. **(B)** Heatmap showing state marker expression in CD8$^+$ T-cell subsets and **(C)** CD8$^+$ T-cell UMAPs coloured by state marker expression. UMAPs represent cells aggregated from both tumour types. GzmB: granzyme B.

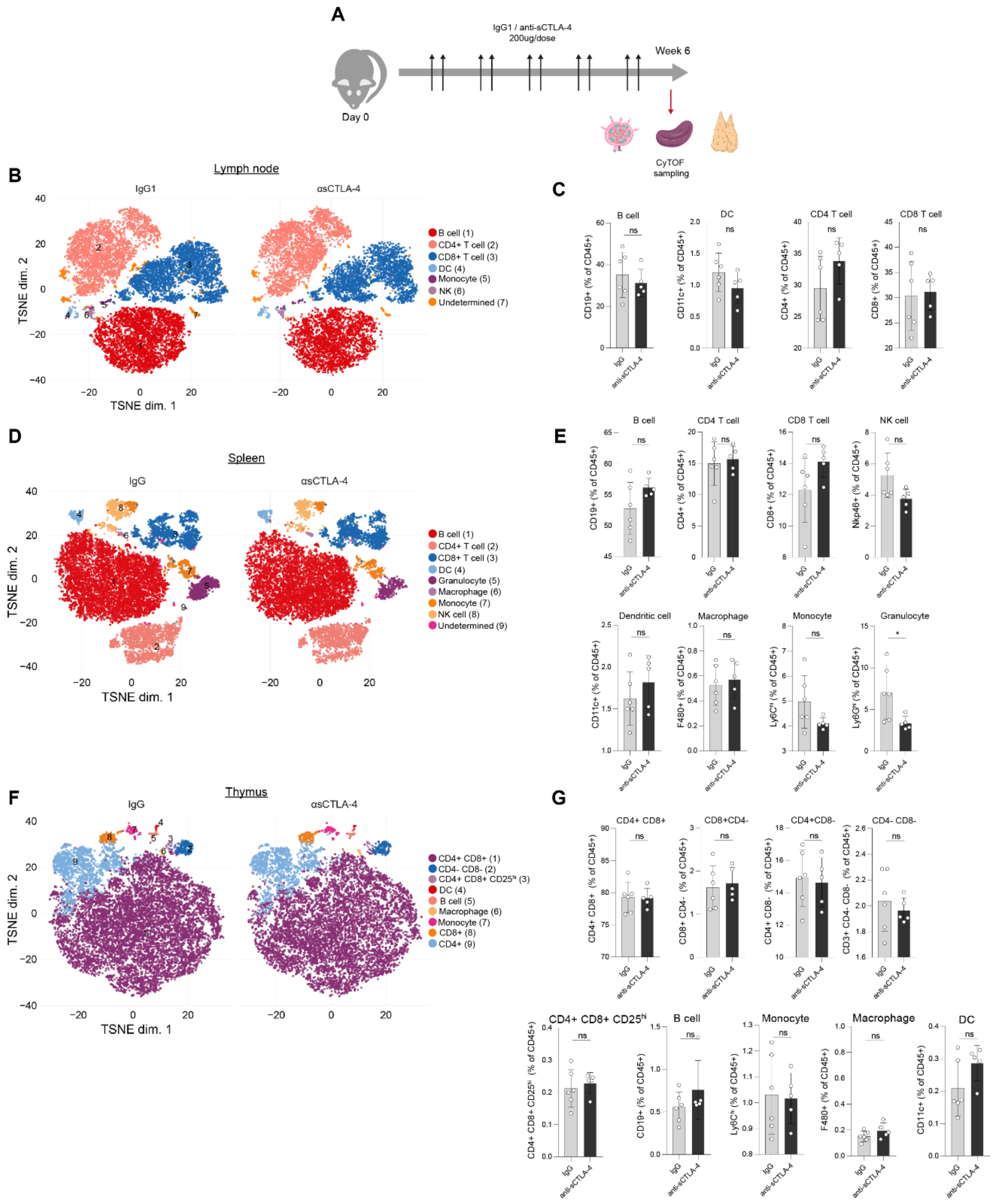

Figure S6. Anti-sCTLA-4 treatment does not perturb immune homeostasis. (A) Five-week-old C57Bl/6 mice were treated twice weekly for 5 weeks with 200ug of anti-sCTLA-4 (JMW-3B3) or Isotype control control antibody. **(B,D,F)** Mass cytometric analysis of mice in a: tSNE analysis showing flowSOM based clustering of major cell populations within lymph node, spleen, and thymus respectively. **(C,E,G)** Quantification of major cell subsets within these organs. Statistical significance was calculated using two-tailed Student's *t*-test. Data are based on aggregated scaled expression, n=4-6 mice. 5,000 cells per tSNE plot are displayed**.**

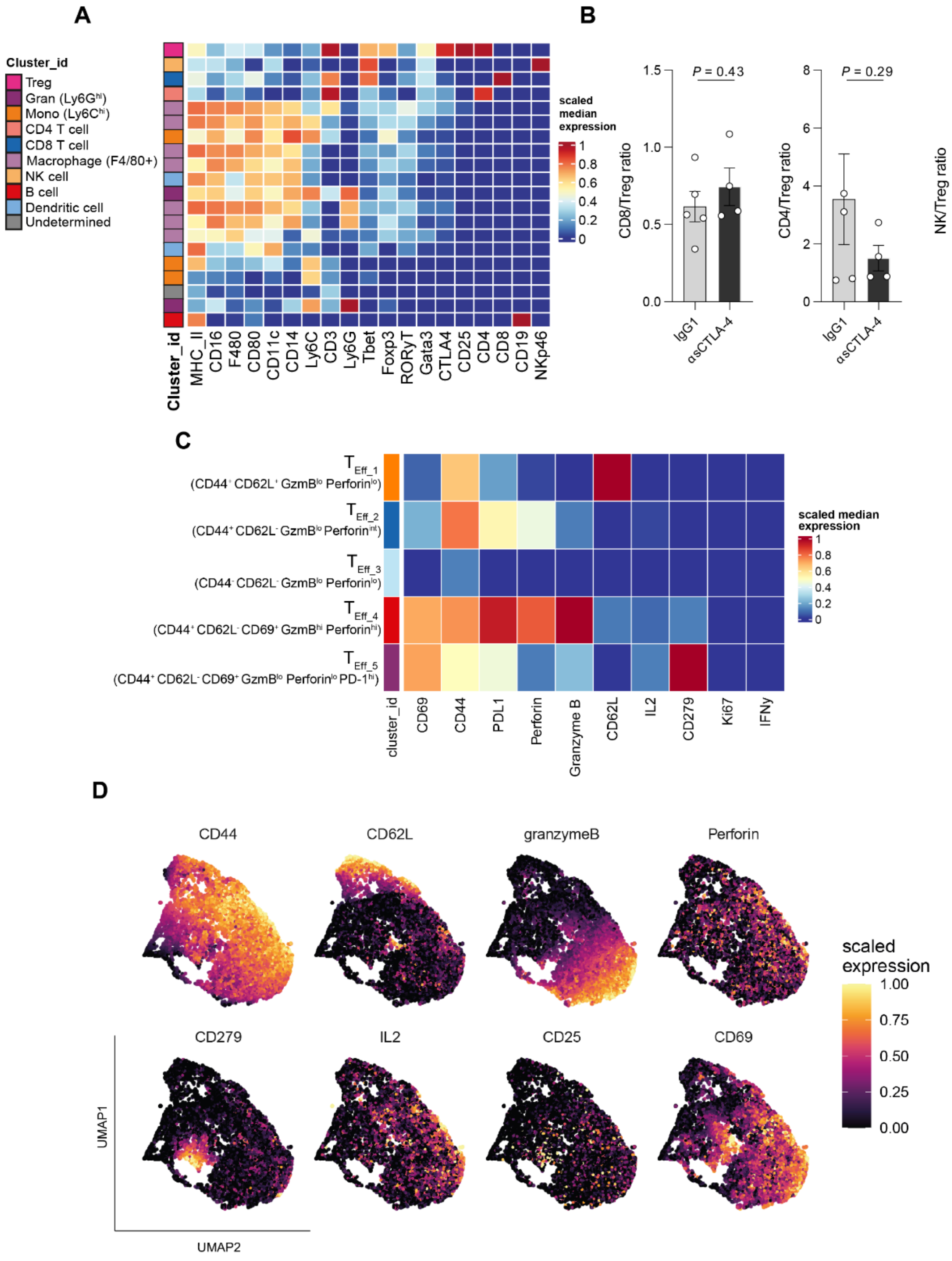

Figure S7. Evaluation of MC38 derived immune infiltrate clusters by mass cytometry. (A) Heatmap showing scaled marker expression in manually annotated clusters from data shown in **Fig. 4C**. 20 flowSOM-identified metaclusters were manually annotated and merged according to lineage marker expression. Over-clustering of the data identified multiple F4/80+ subsets with variable marker expression, which were merged into a macrophage lineage for clarity. **(B)** Lymphocyte: T_{reg} cell ratios of MC38 tumour bearing mice treated with anti-sCTLA-4 or isotype control antibody. Data are expressed as mean ± SD; n = 4 mice per arm. Statistical significance was calculated using two-tailed Student's t-test. **(C)** Scaled $CD8^+$ T-cell marker expression in MC38 tumours with heatmap showing state marker expression in $CD8^+$ T-cell clusters. **(D)** CD8 T cell UMAPs coloured by state marker expression. GzmB: granzyme B.

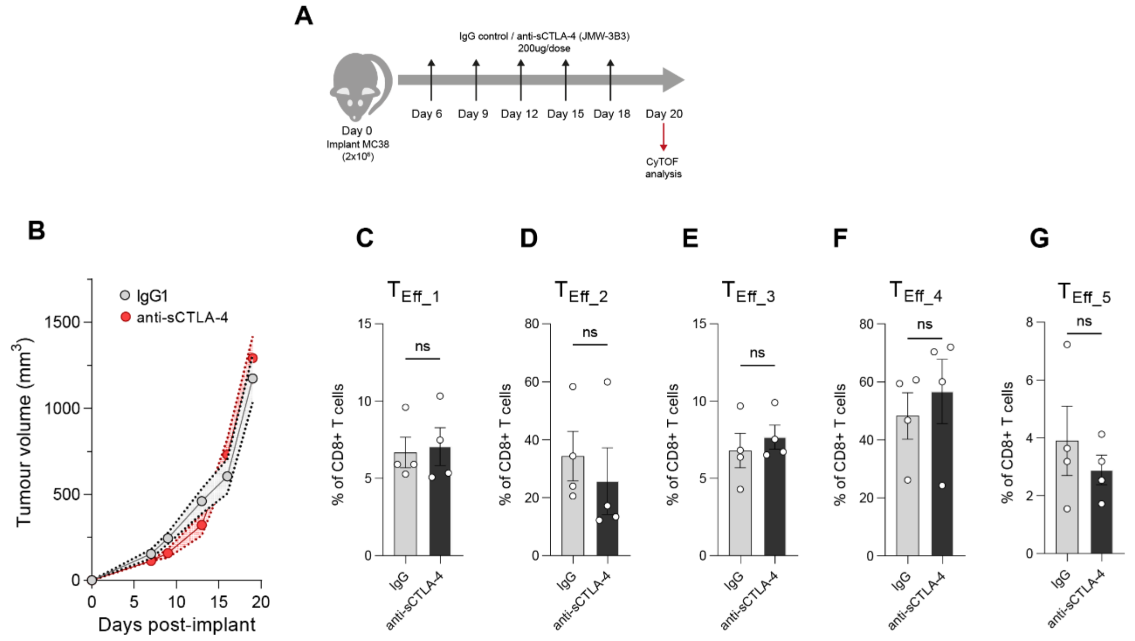

Figure S8. High MC38 tumour burden reduces efficacy induced by anti-sCTLA-4 treatment. (A-B) Mice were injected with high inoculum of MC38 cells ($2x10^6$) and treated with anti-sCTLA-4. Tumours were collected for mass cytometry-based analysis of the infiltrate on day 20 post-inoculation. Data are mean ± SEM, n=4 mice per group. **(C-G)** flowSOM-based metaclustering of $CD8^+$ T cells within MC38 infiltrate. Five clusters identified by flowSOM were manually annotated as T_{Eff_1} ($CD44^+CD62L^+GzmB^-Perforin^-$); T_{Eff_2} ($CD44^+$ $CD62L^+$ Granzyme B^- $Perforin^{low}$); T_{Eff_3} ($CD44^-$ $CD62L^-$ Granzyme B^- $Perforin^-$); T_{Eff_4} ($CD44^+$ $CD62L^-$ Granzyme B^{hi} $Perforin^{hi}$) and T_{Eff_5} ($CD44^-$ $CD62L^-$ Granzyme B^- $Perforin^-$ $PD\text{-}1^{hi}$). (ns $p>0.05$, two-tailed Student's *t*-test). Data are mean ± SEM, n=4 mice per group.

Supplemental tables

Table S1. Antibodies and recombinant proteins used in the study.

Antibody / protein	Clone	Supplier (cat #)	Usage
Anti-β-actin	AC-15	Sigma-Aldrich (A1978)	Immunoblot
Anti-CTLA-4	E1V6T	CST (96399)	Immunoblot
Anti-HA	C29F4	CST (3724)	Immunoblot / IHC
Anti-rabbit IgG, HRP-linked		CST (7074)	Immunoblot
Anti-mouse IgG, HRP-linked		CST (7076)	Immunoblot
Anti-human-CD8-APC	SK1	BioLegend (344722)	Flow cytometry
Anti-human-CD4-APC	SK3	BioLegend (980802)	Flow cytometry
Anti-human-CTLA-4-APC	BNI3	BioLegend (369611)	Flow cytometry
Anti-mouse-CD8a-APC	53-6.7	BioLegend (100711)	Flow cytometry
Anti-mouse-CD4-FITC	RM4-5	BioLegend (100510)	Flow cytometry
Anti-mouse-CTLA-4-APC	UC10-4B9	BioLegend (106309)	Flow cytometry
APC Mouse IgG2a, κ Isotype	MOPC-173	BioLegend (400221)	Flow cytometry
APC Armenian Hamster IgG Isotype	HTK888	BioLegend (400911)	Flow cytometry
CTLA-4-Fc		BioLegend (591802)	Functional study
Anti-sCTLA-4	JMW-3B3	Custom	Functional study
InVivoMAb anti-mouse CTLA-4 (CD152)	9D9	BioXcell (BE0164)	Functional study
InVivoMAb mouse IgG2b	MPC-11	BioXcell (BE0086)	Functional study
InVivoMAb mouse IgG1	MOPC-21	BioXcell (BE0083)	Functional study

Table S2. Mass cytometry antibodies for immunophenotyping.

Target	Conjugate	Antibody clone	Supplier (cat #)
CD45	89Y	30-F11	Standard Biotools (3089005B)
CD45	106Cd	30-F11	Custom (BioLegend 103141)
CD45	111Cd	30-F11	Custom (BioLegend 103141)
CD45	114Cd	30-F11	Custom (BioLegend 103141)
CD45	116Cd	30-F11	Custom (BioLegend 103141)
Ly-6G	141Pr	1A8	Standard Biotools (3141008B)
CD44	142Nd	IM7	Custom (BioLegend 103051)
CD69	143Nd	H1.2F3	Standard Biotools (3143004B)
IL-2	144Nd	JES65H4	Standard Biotools (3144002B)
CD4	145Nd	RM45	Standard Biotools (3145002B)
F4/80	146Nd	BM8	Standard Biotools (3146008B)
CD16	148Nd	S17014E	Custom (BioLegend 158002)
CD25	150Nd	3C7	Standard Biotools (3150002B)
T-bet	151Eu	4B10	Custom (BioLegend 644825)
GATA-3	152Sm	TWAJ	Custom Thermofisher (14-9966-82)
PD-L1	153Eu	10F.9G2	Standard Biotools (3153016B)
CTLA-4	154Sm	UC104B9	Standard Biotools (3154008B)
Perforin	155Gd	OMAK-D	Custom Thermofisher (14-9392-82)
CD14	156Gd	Sa142	Standard Biotools (3156009B)
FoxP3	158Gd	FJK16s	Standard Biotools (3158003A)
RORγT	159Tb	B2D	Standard Biotools (3159019B)
CD3	161Dy	145-2C11	Custom (BioLegend 100345)
Ly-6C	162Dy	HK1.4	Standard Biotools (3162014B)
CD62L	164Dy	MEL14	Standard Biotools (3164003B)
CD19	166Er	6D5	Standard Biotools (3166015B)
NKp46	167Er	29A1.4	Standard Biotools (3166015B)
CD8	168Er	[illegible]36.7	Standard Biotools (3168003B)
CD279/PD-1	169Tm	RMP1-30	Custom (BioLegend 109113)
CD80	171Yb	1610A1	Standard Biotools (3171008B)
MHC II	172Yb	M5/114.15.2	Custom (BioLegend #107637)
GranzymeB	173Yb	GB11	Standard Biotools (3173006B)
CD11c	209Bi	N418	Standard Biotools (3209005B)
